# HPV clearance in postpartum period of HIV-positive and negative women: a prospective follow-up study

**DOI:** 10.1186/1471-2334-13-564

**Published:** 2013-12-01

**Authors:** Emilia Moreira Jalil, Francisco Inacio Bastos, Patricia Pereira dos Santos Melli, Geraldo Duarte, Renata Toscano Simoes, Aparecida Yulie Yamamoto, Rodrigo Augustus Amaral de Morais, Silvana Maria Quintana

**Affiliations:** 1 ICICT/ENSP - Fundação Oswaldo Cruz - FIOCRUZ, Biblioteca de Manguinhos suite 229, Av. Brasil 4365, Rio de Janeiro RJ 21045-900, Brazil; 2 CAPES Visiting scholar, Imperial College London, London, UK; 3 Faculdade de Medicina de Ribeirão Preto, Universidade de São Paulo, Sao Paulo, Brazil; 4 Instituto de Ensino e Pesquisa da Santa Casa de, Belo Horizonte, Brazil

**Keywords:** Human Papillomavirus, HIV, Pregnancy, Postpartum Period, Polymerase Chain Reaction (PCR)

## Abstract

**Background:**

HPV persistence is a key determinant of cervical carcinogenesis. The influence of postpartum on HPV clearance has been debated. This study aimed to assess HPV clearance in later pregnancy and postpartum among HIV-positive and negative women.

**Methods:**

We conducted a follow-up study with 151 HPV-positive women coinfected with HIV, in 2007–2010. After baseline assessment, all women were retested for HPV infection using PCR in later pregnancy and after delivery. Multivariable logistic regressions assessed the putative association of covariates with HPV status in between each one of the successive visits.

**Results:**

Seventy-one women (47%) have eliminated HPV between the baseline visit and their second or third visits. HIV-positive women took a significantly longer time (7.0 ± 3.8 months) to clear HPV, compared to those not infected by HIV (5.9 ± 3.0 months). HPV clearance was significantly more likely to take place after delivery than during pregnancy (84.5% x 15.5%).

**Conclusions:**

Both HIV-positive and negative women presented a significant reduction in HPV infection during the postpartum period. HIV-positive status was found to be associated with a longer period of time to clear HPV infection in pregnant women.

## Background

Human papillomavirus (HPV) is highly prevalent in non-vaccinated sexually active women worldwide, even among those presenting normal cytology [1]. Most HPV infections are transient and self-limited. Up to 90% HPV infections are eliminated after 12–24 months [2-5]. Only 10-15% of women develop a persistent infection, which is one of the most important risk factors for cervical carcinogenesis [6-9]. Even short-time HPV persistence has been associated with higher risk for cervical intra-epithelial neoplasia, compared to women without a history of HPV infection [10,11].

HPV clearance is more frequent in the first six months after prime infection, with rates of 50-70% per follow-up year [5,12,13]. Adequate cell immune response is crucial for HPV clearance [14,15]. On the other hand, immune tolerance/deficiency favours viral persistence and cervical cancer progression [16].

HIV-positive women have a higher risk of acquiring new HPV infections and a lower chance of clearing them compared to women not infected by HIV [17-21]. Markers of cell immunity system, e.g. higher CD4+ T cell counts, have been associated with HPV clearance in HIV-positive women [21,22].

Changes in immunity and other biological parameters (e.g. changes in the levels of different hormones) during pregnancy and postpartum may modulate the natural history of HPV infection. Some recent studies did not observe differences in HPV status over time, during pregnancy [23-25]. Most authors, however, have found a reduction in HPV positivity during the postpartum period [26-29]. Minkoff et al. [30] evaluated HIV-positive women and documented higher numbers of new HPV types at the postpartum period than during pregnancy, but their study did not asses HPV clearance.

The dynamics of HPV infection during pregnancy is not well established and information remains scarce and controversial. Few studies have studied HPV clearance and persistence during and after pregnancy, and just one of them has prospectively evaluated HPV infection in HIV-positive women. In this study, we aimed to evaluate HPV clearance in the postpartum period of both HIV-positive and negative women.

## Methods

A prospective follow-up study was carried out between January 2007 and January 2010, in Ribeirão Preto, São Paulo state, Brazil. Pregnant women were selected consecutively from the Prenatal Outpatient Clinic of the Infectious Diseases Unit, Obstetrics and Gynecology Department of the University Hospital, Medical School of Ribeirão Preto, University of São Paulo. This is a tertiary service, which follows-up with women previously diagnosed with HIV or HPV infection at primary units, selected from a large catchment population mainly composed of women from middle and low socioeconomic strata. Women were evaluated according to their HIV status and/or HPV-related lesion. HPV infection type was classified as clinical (in case of warts), subclinical (in case that colposcopic lesions were made evident), or HPV-positive (if only PCR-positive).

Inclusion criteria were as follows: 1) be in the 1st half of pregnancy; 2) be positive for HPV, as defined by a PCR exam; 3) be an adult (≥18 years old; so in full capacity to consent or not to take part of the study according to Brazilian law); and 4) have read and signed the informed consent form.

Cervicovaginal samples (with 2 mL of saline solution) were collected from all women and tested for HPV by Polymerase Chain Reaction (PCR). Women who tested positive for HPV were re-evaluated at two subsequent visits: at the 2nd half of pregnancy and after delivery.

The study protocol was approved by the Ethics Review Board of the Ribeirao Preto’s Medical School Hospital – Sao Paulo University, and all women voluntarily signed informed consent before enrolment. All patients followed antenatal care routine of the service and no procedure was altered for those women enrolled in the study, except for the three PCR exams performed in each one of the visits (during that period, PCR was not a routine exam in the outpatient clinic).

Cervicovaginal samples were stored at -80°C until DNA extraction, which was performed by QIAamp® DNA Mini Kit (Qiagen, USA). Consensus primers GP5+ / GP6+ were used to identify HPV positivity, as previously described [31]. After that, HPV types 6 and 11, considered low-risk HPV (LR-HPV) and 16, 18, 31 and 33, high-risk HPV (HR-HPV), were identified simultaneously by multiplex PCR using specific primers [32]. Every single woman had her HPV infection typed at least once. Primers S-GH20, pCO3 and pCO4 were used as internal controls [32,33]. Negative controls were included in every single step in order to minimize false positive results; positive controls used were known positive samples collected in previous studies and belonging to a panel of positive and negative controls.

Sample size was calculated with the help of Graphpad StatMate® (Graphpad Software, USA). Assuming a 15% differential clearance over time (from early pregnancy up to postpartum), it would be necessary to include at least 99 patients, considering an 80% power and an alpha of 5%.

HPV clearance was defined as having at least one negative result after a confirmed positive one. A negative test detected in the 2nd visit (followed by a negative one in the 3rd visit) was defined as “clearance”, taking place between the 1st and the 2nd visit (i.e. clearance during pregnancy). Women with positive tests in the first and second visits, followed by a negative test in the 3rd visit, were defined as “clearance” occurring between the 2nd and the 3rd visits (i.e. clearance during the postpartum period).

Persistent infection was defined for the sake of our study as “the consecutive detection of at least two positive HPV tests between the second and the third visit” (considering that all women tested positive at baseline). When a negative result appeared in between two positive tests (i.e. between the baseline and the third visit), the intermediate result was defined as a false negative, in agreement with previous studies and their respective criteria [34,35]. Duration of infection was estimated as the period (in months) elapsed between the baseline visit and a mid-point between the baseline visit and the first negative result, in all cases where no positive result followed (i.e. a false negative).

Statistical analysis was carried out with the help of EpiInfo 3.5.3 (CDC, USA) and SAS 9.0 (SAS Institute, USA). Paired t-tests (with Welch correction whether unequal variances were made evident) and chi-square test (or Fisher Exact test for table cells with less than 5 events) were used for univariate analysis. Multiple log-binomial regression models were used, as the outcome was binary; procedure PROC GENMOD was applied for model adjustment. Multivariable analyses were controlled for participants’ age, smoking/non-smoking, mode of delivery, HIV status, nature of HPV infection (clinically defined or otherwise), and HPV type (dichotomous variable: high vs. low risk).

## Results

**Figure 1:**
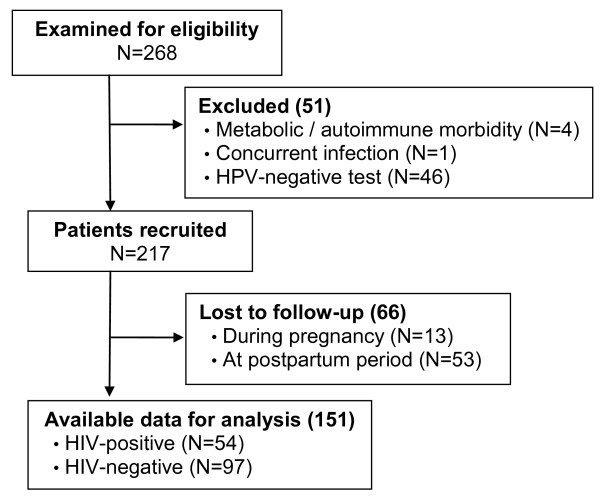
Flow diagram of study population.

One hundred and fifty-one HPV-positive women were enrolled and followed-up with, of which 54 were HIV-positive and 97 were HIV-negative (Figure 1). Mean gestational age at entrance and at 2nd visit were 13.1 ± 4.2 and 32.6 ± 5.0 weeks, respectively. The mean visit interval did not differ significantly among HIV-positive (5.47 ± 2.6) and negative (5.0 ± 2.1 months) women. Mean interval between the 1st and the 2nd visit was 17.1 ± 6.3 weeks and postpartum visit occurred 4.7 ± 2.2 months after delivery. On average, HIV-positive women were older than HIV-negative women (28.6 ± 6.1 years vs. 23.2 ± 6.4 years) and had a longer follow-up period (9.6 ± 3.2 months vs. 7.8 ± 2.9 months). Most women had vaginal deliveries (53.7% of HIV-positive and 62.0% of HIV-negative women). HIV-positive women had a higher proportion of infections which were exclusively detected by PCR, compared to their HIV-negative counterparts (Table 1).

**Table 1:** Baseline characteristics of 151 HPV-positive women under follow-up, according to HIV status

**Characteristics**	**HIV-positive women**		**HIV-negative women**	
	**N = 54**	**%**	**N = 97**	**%**
**Marital status**				
Non-stable	16	29.6	35	36.1
Stable	38	70.4	62	63.9
**Race/skin color**				
White	26	48.2	68	70.1
Brown	18	33.3	19	19.6
Black	10	18.5	10	10.3
**Years of education**				
0-4	7	13.0	13	13.4
5-8	24	44.4	37	38.1
>9	23	42.6	47	48.4
**Menarche (years)**				
<9	2	3.7	2	2.1
9-15	48	88.9	87	89.7
>15	4	7.4	8	8.2
**Age at 1st sexual experience (years)**				
<16	30	55.6	63	64.9
16-20	22	40.7	31	32.0
>20	2	3.7	3	3.1
**Age of 1st pregnancy (years)**				
<16	13	24.1	20	20.6
16-20	23	42.6	51	52.6
>20	18	33.3	26	26.8
**Parity**				
0	11	20.4	66	68.0
1-3	38	70.4	30	30.9
≥4	5	9.2	1	1.1
**Lifetime no. of sex partners**				
≤5	31	57.4	74	76.3
6-10	12	22.2	12	12.4
11-20	6	11.1	8	8.2
>20	5	9.3	3	3.1
**Sex partners (last 12 mos.)**				
<3	51	94.4	92	94.8
≥3	3	5.6	5	5.2
**Soking**	19	35.2	18	18.6
**Drug use**				
None	41	75.9	77	79.4
Marijuana	9	16.7	15	15.5
Cocaine/crack	4	7.4	5	5.1
**Alcohol**	9	16.7	11	11.3
**Previous STD**	37	68.5	17	17.5
**Sexual partner with STD**^**1**^	18	33.3	13	13.4
**HPV infection type**				
HPV-positive	32	59.3	8	8.3
Subclinical	22	40.7	62	63.9
Clinical	0	0	27	27.8

Ribeirão Preto, 2007-2010.

^1^Referred by women.

Overall genotype prevalences were as follows (in descending order of frequency): 37.1% (HPV 6 / 11), 23.8% (HPV 18), 13.2% (HPV 16), 4.0% (HPV 31), and 4.0% (HPV 33). None of these genotypes were identified in 43 samples (28.5%). The socio-demographic characteristics, as well as clinical and laboratory data did not significantly differ between these women and those form whom HPV infection was typed.

More than half of the HIV-positive women had their condition diagnosed less than a year ago (28/54). More than 60% of these women had CD4+ counts above 350 cells/mm^3^ and 44% of them present viral loads above 10,000 copies/mL.

Overall, clearance rate was 47% (71/151). The groups among whom HPV had persisted or cleared did not differ in median age (25.8 ± 7.5 years vs. 24.4 ± 6.0 years; p = 0.2), nor in gestational age at their 1st visit (17.3 ± 6.8 weeks vs. 17.0 ± 7.7; p = 0.87). HIV-positive and negative women did not significantly differ regarding HPV clearance rates (48.1% vs. 46.4%; p = 0.87); but HIV-positive women took a significantly longer time (7.0 ± 3.8 months) to clear HPV, compared to those not infected by HIV (5.9 ± 3.0 months).

Univariate analyses are summarised in Table 2. HPV clearance was found to be associated with the following covariates: “to be engaged in a stable marital relationship”, “to be white” (versus brown [biracial] and black), and “no previous engagement in commercial sex”. “Mode of delivery”, “presentation of HPV infection” (clinical/subclinical/HPV-positive) and “HPV type” were not found to be associated with viral clearance. The group of women among whom HPV has persisted showed a higher percentage of cytological abnormalities compared to the group composed by women among whom HPV was cleared (67.5% vs. 40.8%; p = 0.002).

**Table 2:** Characteristics associated with HPV clearance among 151 HPV-positive women (univariate analysis)

**Characteristics**	**Persistence**		**Clearance**		***p***
	**N = 80**	**%**	**N = 71**	**%**	
**Marital status**					
Non-stable	34	42.5	17	23.9	<0.05*
Stable	46	57.5	54	76.1	
**Race/skin color**					
White	40	50.0	54	76.1	**<0.05**^#^
Brown	24	30.0	13	18.3	
Black	16	20.0	4	5.6	
**Years of education**					
0-4	13	16.2	7	9.9	0.68^#^
5-8	31	38.8	30	42.2	
>9	36	45.0	34	47.9	
**Menarche (yrs.)**					
<9	3	3.75	1	1.4	0.09^#^
9-15	74	92.5	61	85.9	
>15	3	3.75	9	12.7	
**Age at 1st sexual experience (yrs.)**					
<16	54	67.5	39	54.9	0.19^#^
16-20	23	28.8	30	42.3	
>20	3	3.7	2	2.8	
**Age of 1st pregnancy (yrs.)**					
<16	22	27.5	11	15.5	0.17*
16-20	38	47.5	36	50.7	
>20	20	25.0	24	33.8	
**Parity**					
0	40	50.0	37	52.1	0.83^#^
1-3	38	47.5	30	42.3	
≥4	2	2.5	4	5.6	
**Lifetime no. of sex partners**					
≤5	50	62.5	55	77.5	0.18^#^
6-10	14	17.5	10	14.1	
11-20	10	12.5	4	5.6	
>20	6	7.5	2	2.8	
**Sex partners (last 12 mos.)**					
<3	75	93.8	68	95.8	1.0^#^
≥3	5	6.2	3	4.2	
**HIV-positive status**	28	35.0	26	36.6	0.84*
**Smoking**	22	27.5	15	21.1	0.36*
**Drug use**					
None	59	73.8	59	83.1	0.39^#^
Marijuana	14	17.5	10	14.1	
Cocaine/crack	7	8.7	2	2.8	
**Alcohol**	15	18.8	5	7.0	0.053^#^
**Previous STD**	30	37.5	24	33.8	0.64*
**Sexual partner with STD**^**1**^	18	22.5	13	18.3	0.52*
**Mode of delivery**					
Cesarean section	37	46.2	27	38.0	0.33
Vaginal	43	53.8	44	62.0	
**HPV infection type**					
Subclinical/clinical	60	75.0	51	71.8	0.66
HPV-positive	20	25.0	20	28.2	
**HPV type**					
High risk	34	64.1	22	54.4	0.3
Low risk	19	35.9	20	47.6	

Ribeirão Preto, 2007-2010.

*Chi-square test; ^#^Fisher Exact Test; ^1^Referred by women.

Clearance rate was slightly higher in women with LR-HPV than those with HR-HPV infection, but such difference was not found to be statistically significant (20/39 (51.3%) vs. 22/56 (39.3%); p = 0.25).

Almost half of the women living with HIV infection cleared HPV infection during follow-up (26/54). Lower HIV viral load and higher CD4+ cell counts were found to be associated with HPV clearance. More than 75% of HIV-women with CD4+ count over 350 cells/mm^3^ cleared HPV during follow-up (20/26) and only 4 women with less than 1,000 copies/mL had a persistent HPV infection over the follow-up period (4/11).

In multivariable analysis, non-smoker women had a two-fold higher chance to clear HPV (RR 2.2; IC95% 1.1–5.0) compared with smokers. Additional data are presented in Table 3. The association between CD4+ cell count and HPV clearance did not remain statistically significant after controlling for other covariates (RR 2.0 [0.6-6.3])].

**Table 3:** Characteristics associated with HPV clearance among 151 HPV-positive women (multivariable analysis)

**Variables**	Adjusted RR (CI95%)*
**Age (years)**	
>25	1
≤25	0.9 (0.5-1.5)
**Smoking**	
Yes	1
No	2.2 (1.1-5.0)
**Mode of delivery**	
Cesarean section	
Vaginal	1.1 (0.6-1.9)
**HIV status**	
Positive	1
Negative	1.0 (0.6-1.7)
**Nature of HPV infection**	
Subclinical/clinical	1
HPV-positive	1.4 (0.7-2.7)
**HPV type**	
High risk	1
Low risk	1.4 (0.9-2.3)

Ribeirão Preto, 2007-2010.

RR: Relative risk; CI95%: Confidence interval 95%.

*Adjusted for all variables listed in table.

HPV clearance was made evident between the 2nd and the 3rd (postpartum) visits in 84.5% of women who cleared HPV infection (60/71), compared to 15.5% during the 1st and 2nd visits (antenatal care) (11/71). Such difference was statistically significant for women, irrespectively of their HIV status, as well as when analyses were stratified by HIV status (Table 4). More than 70% of HIV-negative and 90% of HIV-positive women cleared HPV during the postpartum period.

**Table 4:** HPV clearance during pregnancy and postpartum period (overall and according to HIV status)

**Period of clearance**	**All women**			**HIV-positive women**			**HIV-negative women**		
	**(N = 71)**			**(N = 26)**			**(N = 45)**		
	**N**	**%**	**IC95%**	**N**	**%**	**IC95%**	**N**	**%**	**IC95%**
**Pregnancy**	11	15.5	8 - 26	7	26.9	12 - 48	4	8.9	3 – 22
**Postpartum period**	60	84.5	74 - 92	19	73.1	52 - 88	41	91.1	78 -97

## Discussion and conclusions

Overall clearance rate in this study was 47%, which is in agreement with previous findings, among both pregnant [27,36] and non-pregnant women [37-39]. In a previous Spanish study [36], pregnant women at high-risk of HPV exposure had a clearance rate up to the post-partum visit (53.8%) roughly comparable to our findings. Divergent findings could be due to the analysis of populations with different composition in terms of their genetic background, nutritional and health status, as well as longer or shorter follow-up periods. The very definition of viral clearance has been varied in different studies. Banura et al. [24] identified higher clearance rates in primiparous women from Uganda than those made evident by our study, but their study differs from ours due to the fact that the vast majority of women recruited by them did not have any other medical conditions. Another difference between our studies is that the study in Uganda computed the number of actual infections cleared (discriminated by viral subtypes) instead of the number of women who cleared HPV infection.

High HPV clearance rates between 68.3 and 80.7% have been reported among non-pregnant women without cytological abnormalities, under a longer follow-up period [13,35,40,41]. On the other hand, Banura et al. [42] documented viral clearance in 31.2% of Ugandan non-pregnant women. The authors considered that the low clearance rate made evident by their study was, in part, due to new infections acquired by subjects during the intervals between subsequent visits.

The current study identified a higher HPV clearance rate during the postpartum period for both HIV-positive and negative women, compared to the period covered by the antenatal visits. These findings corroborate the results previously reported by Nobbenhuis et al. [27], who found lower HPV positivity in women with abnormal cytology after delivery, compared to clearance observed over all gestational trimesters. Data eventuating from a re-analysis of a German cohort documented a higher risk for HPV infection in pregnant vis-à-vis puerperal women [28].

Cervical trauma during delivery may be associated with lower HPV-infection rates during postpartum. Previous studies identified higher regression rates of HPV-related lesions among women who delivered vaginally compared to those who had Caesarean sections [43,44], in contrast to other studies, which found similar regression rates for women who had vaginal or c-section deliveries [45,46]. Ours is the first study to assess HPV clearance in the context of different modes of delivery, but our findings did not make evident any significant difference between them respecting HPV clearance. (This may be, however, due to the lack of statistical power to fully explore such hypothesis).

More than 70% of HIV-positive women enrolled in the study cleared HPV over time, a higher rate than has been previously reported among non-pregnant women. Denny et al. [47] observed an HPV clearance of 6% of South-African non-pregnant women living with HIV, over an 18-month follow-up period. An Italian study reported a 22.8% HPV clearance rate in HIV-positive women over 14 months [19]. Our favourable evolution might therefore be secondary to good immunologic status or relatively low frequency of cytological abnormalities in HIV-positive women enrolled in our study.

Brazilian health care system guarantees the full access to free antiretroviral therapy to any eligible patient according to criteria which are similar to the updated criteria issued by the World Health Organization (WHO). More than 90% of our patients have used this therapy during pregnancy, which might influence our results. A recent study has demonstrated that highly active antiretroviral therapy may increase the clearance of some oncogenic HPV types [48]. These factors may have confounded the association between HIV status and HPV persistence.

Ours is the first study to assess HPV clearance among HIV-positive women during pregnancy and postpartum. Minkoff et al. [30] compared HPV prevalence and incidence in pregnancy and puerperium in HIV-positive women. The authors identified a higher incidence rate for HPV infection in the postpartum period.

In the HER Study (HIV Epidemiology Research), which included more than 500 HIV-positive women, higher CD4+ cell counts were found to be associated with a higher HPV clearance [22]. Rowhani-Rahbar et al. [21] identified that HPV clearance was inversely proportional to HIV viral load and less likely to occur in women with lower CD4+ cell count, especially among those with CD4+ counts bellow 200 cells/mm^3^. A South-African study found that low CD4+ count was associated with a higher HPV infection incidence, but no association with clearance was made evident by it. Clearance was found to be exclusively associated with HIV viral load [47]. Furthermore, it has been suggested that HIV-HPV coinfections have a broader impact on immunity, beyond their impact on CD4+ count [21]. In the current study, CD4+ cell count was not found to be associated with HPV clearance.

We further analysed the database in order to assess a putative linear relationship between successive categories of CD4+ counts (<200, 200–350, 350–500, 500+) and HPV clearance (data not shown). Unfortunately, we did not observe any gradient, either because it does not exist or because of our small sample size (beta error).

The study assessed HPV types using a modest set of primers and HPV type-persistence was not assessed. Further studies, profiting from a comprehensive variety of HPV primers and a follow-up of HPV-types incidence/clearance over time is sorely needed in order to complement and validate our findings.

In longitudinal studies, clearance rates and median infection duration might be underestimated due to left-censoring of infections present at the beginning of the study and right-censoring of infections that have not been cleared until the end of follow-up. The current study had a relatively short follow-up and intervals between visits, which were limited to pregnancy and post-partum. Such a short period of time (secondary to the logistic of the referral service) might compromise our ability to assess HPV evaluation. Women might have cleared their infection between visits, and it is not possible to specify the precise moment during which clearance may take place. The association between HPV and HIV coinfection might be underestimated in which case a longer follow-up period would be necessary to properly assess it over time.

Our findings should not be generalised to other groups of pregnant women, due to its small sample size, short follow-up time, and the fact that our study enrolled women from a tertiary referral care unit. Other sexually transmitted infections were not investigated such as *Chlamydia trachomatis*. Therefore, some unmeasured confounding may have affected the adjusted risk ratios.

HPV pathophysiology is especially complex due to the impossibility to fully differentiate persistent infections from those which might have occurred during the interval of two consecutive visits. Besides such caveat, a negative HPV test may not represent the full elimination of the virus, since latent infection may remain elusive from the perspective of both clinical and laboratory assessments. According to Schiffman et al. [49], HPV reappearance after a period of latency may be common, even in the absence of immunosuppression. These controversial aspects, combined with the transient nature HPV infection may interfere on any assessment of the natural history of HPV infection.

The current study aimed to better assess some controversial aspects of HPV’s natural history. Postpartum period appears to be a period of significant decline of HPV infection, among both HIV-negative and positive women. The mode of delivery does not seem to influence this decline. A better understanding of HPV dynamics may help to identify women at higher risk of developing HPV-related lesions during antenatal care, contributing to prevention of cervical cancer, especially among HIV-positive women, among whom such cancers tend to be especially aggressive and harmful.

## Abbreviations

HPV: Human papillomavirus; HIV: Human immunodeficiency virus; PCR: Polymerase chain reaction; LR-HPV: Low-risk HPV; HR-HPV: High-risk HPV.

## Competing interests

The authors declare that they have no competing interests.

## Authors’ contributions

EMJ and SMQ conceived and designed the project, and analysed data. PPSM participated in data collection. GD and FIB provided guidance on this project. RTS and AYY carried out the PCR analysis. RAAM contributed to the data analysis and in the review process. All authors have been involved in drafting or revising the manuscript and have approved its final version.

## Pre-publication history

The pre-publication history for this paper can be accessed here:

http://www.biomedcentral.com/1471-2334/13/564/prepub
